# Ionic Conductivity and Structure of Chitosan Films Modified with Lactic Acid-Choline Chloride NADES

**DOI:** 10.3390/polym12020350

**Published:** 2020-02-06

**Authors:** Mikhail A. Smirnov, Alexandra L. Nikolaeva, Vitaly K. Vorobiov, Natalia V. Bobrova, Ivan V. Abalov, Alexander V. Smirnov, Maria P. Sokolova

**Affiliations:** 1 Institute of Macromolecular Compounds Russian Academy of Sciences, Bolshoy pr. 31, Saint Petersburg 199004, Russia; Smirnov_Michael@mail.ru (M.A.S.); alexandra.l.nikolaeva@gmail.com (A.L.N.); vrbvrbvrb@mail.ru (V.K.V.); bobrovanatalialab19@mail.ru (N.V.B.); i.abalf@yandex.ru (I.V.A.); 2 Physics and Technology Faculty, ITMO University, Kronverskii prosp. 49, Saint Petersburg 197101, Russia; smirnav_2@mail.ru; 3 Saint Petersburg State University, Institute of Chemistry, Universitetskaya nab. 7-9, Saint Petersburg 198504, Russia

**Keywords:** chitosan, natural deep eutectic solvent, polymer electrolytes, plasticization

## Abstract

The natural deep eutectic solvent (NADES) based on choline chloride (ChCl) and lactic acid (LA) was used for the preparation of chitosan (CS) films by the solution casting method. The content of NADES in films was from 0 to 82 wt%. The impact of NADES on the morphology and crystalline structure of films was investigated using scanning electron microscopy as well as wide-angle and small-angle X-ray scattering. The experimental results allow to propose CS chains swelling in NADES. FTIR spectroscopy confirms the interactions between CS and NADES components via the formation of hydrogen and ion bonds. The thermal properties of the composite films were studied by simultaneous thermogravimetric and differential thermal analysis. Thermomechanical analysis demonstrated appearance of two transitions at temperatures between −23 and −5 °C and 54–102 °C depending on NADES content. It was found that electrical conductivity of film with 82 wt% of NADES reaches 1.7 mS/cm. The influence of the composition and structure of films on the charge carriers concentration and their mobility is discussed.

## 1. Introduction

Chitosan (CS) is a linear copolymer composed of β-(1-4) linked 2-acetamido-2-deoxy-*β*-D-glucopyranose and 2-amino-2-deoxy-β-D-glycopyranose obtained by deacetylation of chitin. Necessity in replacement of artificial polymers with renewable ones leads to the intensive study of CS and chitin for various applications including drug delivery vehicles [1], tissue engineering [2], water treatment [3], pervaporation membranes [4], food packaging [5] and energy storage devices [6,7]. The solid polymer electrolytes based on natural polymers have received significant attention during recent years [8] due to their environmental safety and low production cost. Biopolymers such as CS and cellulose were used for preparation of ionically conducting membranes [9], separators for electrical double-layer capacitors [10,11], Li-ion batteries [12], thermopower generators [13] and supercapacitor electrodes [14]. Polymer gel electrolytes containing ionic liquids (ILs) are of special interest [15] because ILs have low vapour pressure and provide wide electrochemical stability window to the material. In addition, it was reported that, due to high ion concentration, ILs are ideal electrolytes for accumulation of large charge carrier densities at low voltages. This makes them promising for application in organic field-effect transistors based on polymer electrolytes [16]. Another prospective field for the application of ion gels is the electrical transducers for the measurement of electrical signals of biological objects, such as cardiogram measurements [17].

Thus, the development of preparation methods for polymer-IL gel electrolytes with natural polymers is a significant task for modern material science. The finding of an appropriate volatile cosolvent for IL and polymer is a prerequisite for easy preparation of ion gel membranes. Water is the most simple and green solvent which can be used for this purpose. However, in the case of gelation of IL with cellulose even small amount of water (> 15 wt%) can lead to a decrease in cellulose compatibility with butyl-methyl-imidazolium chloride [18]. This problem was avoided by using of cellulose derivatives, for example, the ion-conducting cellulose triacetate membrane containing *N*-methyl-*N*’-propylpyrrolidinium bis(trifluoromethanesulfonyl) imide and the lithium salt with conductivity up to 0.923 mS/cm was reported [19] (acetone and CH_2_Cl_2_ were used as a solvents for casting of films). In this sense, the CS is an attractive alternative for cellulose, because it was demonstrated, that CS based electrolytes can be prepared from a mixed aqueous solution with 1-ethyl-3-methylimidazolium tetrafluoroborate (EMIm BF_4_) with the subsequent treatment with non-solvent (methanol) [20]. It was pointed out that the resulting ionically conducting films demonstrated suitable mechanical properties: up to 72 MPa Young’s modulus and about 37% elongation at break [20]. CS based gel electrolytes were prepared from an aqueous solution of polymer and acetic or adipic acid and LiCl. After precipitation with NaOH and dehydration by treatment with ethanol, the film was soaked in 1-butyl-3-methylimidazolium tetrafluoroborate (BMIm BF_4_) [21]. The prepared ion gel electrolytes demonstrate electrical conductivity up to 2.9 mS/cm. The application of biodegradable synthetic polymers for the preparation of solid electrolytes was also reported: ion gels based on poly(ε-decalactone)-*b*-poly(DL-lactide)-*b*-poly(ε-decalactone) and a low toxicity ionic liquid, 1-butyl-1-methylpyrrolidinium bistrifluoromethanesulfonylimide were used for a printed electrolyte-gated organic transistor [22].

The wide application of ILs is held back by their high cost, evidences for their toxicity and lack of biodegradability [23,24]. In this sense increased attention is drawn toward alternative liquids with similar physico-chemical properties, i.e., deep eutectic solvents (DES) [25,26,27], and especially their subclass: natural deep eutectic solvents (NADES) [28]. NADES are composed of two or more natural components, which interact via strong hydrogen bonding with formation of a liquid with the melting temperature much lower than those for individual components. One of the widely used acceptors of hydrogen bonds used for the preparation of NADES is choline chloride (ChCl), a cheap and ubiquitous food additive for animals. In the case of NADES, the role of hydrogen bond donor is usually played by natural molecules as urea, polyols or carboxylic acids, such as citric or lactic acid (LA).

The introduction of DES into the polymer matrix opens up a possibility of combining the physico-chemical properties of ILs with the mechanical properties of polymers in green materials. For example, ternary NADES based on ChCl, urea, and glycerol were used for the preparation of ion-gel electrolyte with phthaloyl starch, and the maximal conductivity of 2.86 mS/cm for this system was reported [29]. Ion gels containing ChCl were elaborated using 2-cholinium lactate methacrylate polymerized in the presence of LA-ChCl DES for application as a signal transducers for electro-cardiogram measurements [17]. However, the formation of carbon-based polymer main chain does not allow biodegradation of these materials. The preparation of materials based on CS with LA and ChCl was reported and mechanical properties, water sorption and permeability for the materials containing 30 wt% of this NADES were studied [30]. The acetic acid solution (3%) was used for mixing of CS and NADES and hot-pressing was applied for formation of films. An additional benefit, which is especially important for polysaccharides is a plasticizing effect of DES [31,32], which gives potential for obtaining of thermoplastic and elastic materials based on starch [33] and chitosan [34,35]. These polymers in pure state cannot be processed via common methods of polymer industry like extrusion or thermo-compression molding. The preparation of DES-based composites is considered as a possibility to solve this problem [36].

However, to the best of our knowledge, the electrochemical properties of CS-LA-ChCl plasticized films prepared via solution casting were not studied yet. It was reported recently that NADES based on ChCl and LA (1:1 mole:mole) demonstrate ionic conductivity 0.2–10 mS/cm depending on water content [37]. Thus, it can be presumed, that introduction of this NADES into CS is interesting for the preparation of ionically conducting films. The additional benefit is that this material can be prepared by a simple solution casting method due to the possibility of dissolution of CS in water containing LA if an appropriate amount of acid is used. The aim of this work was to prepare CS films containing ChCl and LA and to investigate the influence of their composition on the ionic conductivity, structure, mechanical, and thermal behavior of obtained materials.

## 2. Materials and Methods

### 2.1. Materials

DL-Lactic acid (LA, 2-Hydroxypropionic acid, 80% (*w*/*w*), Neva Reaktiv, Saint Petersburg, Russia) was used as received without purification. Choline chloride (ChCl, (2-hydroxyethyl) trimethylammonium chloride) was obtained from AppliChem (≥ 98 wt%, Darmstadt, Germany). The ChCl was dried under vacuum before used. CS (with a *M_w_* = 900,000) was purchased from “Bioprogress CJSC” (Schelkovo, Russia) and used without further purification. The degree of deacetylation, %DA = 68.2% [35].

### 2.2. Films Preparation

CS, ChCl powders and LA were weighed and mixed in a 100 mL flask. The mass of CS was taken as 1-(ω(DES)/100%) g, where ω(DES) is the wt% of NADES relative to the overall mass of the film (CS + NADES). The masses of LA and ChCl were taken so as to maintain 1:2 mole:mole (ChCl:LA) ratio between the components of NADES. The following set of ω(NADES) was used: 50, 67, 75 and 82 wt%. The complete dissolution of CS was attained only for 50 wt% NADES content or higher. The water (48 mL) was added to the flask, and the dissolution proceeded under stirring at room temperature (20–25 °C) for 48 h. At this stage, LA provided an acidic medium, which is necessary for the dissolving of CS. The obtained solutions were filtered and degassed under vacuum. The overall concentration of polymer, LA and ChCl in casting solution was maintained equal to 2%.

**Figure 1:**
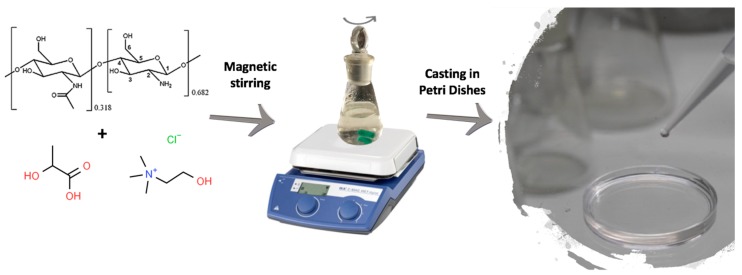
Schematic illustration of the preparation of chitosan-lactic acid-choline chloride (CS-LA-ChCl) films.

Films with the thickness of 20 μm were prepared by casting solutions on Petri dishes (Figure 1) with subsequent drying at 25 °C for 6 days. After being dried, the films were peeled off. The films prepared with NADES contents: 50, 67, 75 and 82 wt% are denoted in the text as CS/DES-50, CS/DES-67, CS/DES-75 and CS/DES-82, respectively. The reference CS/DES-0 film was prepared by a similar procedure but without additional ChCl. All films were colourless and transparent.

### 2.3. Characterization Methods

#### 2.3.1. Fourier Transform Infrared Spectroscopy

Fourier transform infrared (FTIR) spectroscopy study was performed on the IRAffinity-1S spectrometer (Shimadzu, Kyoto, Japan) with 100 scans at resolution 2 cm^−1^ from 2000 to 600 cm^−1^. Spectrum of CS was measured using KBr pellet, while films were investigated in the transmission mode.

#### 2.3.2. Microscopic Investigation

Scanning electron microscopy (SEM) micrographs of the films’ surfaces and cross-sections were obtained with a Zeiss AURIGA Laser microscope (Carl Zeiss, Oberkochen, Germany) at 1 kV voltage. For investigation of cross-sections, the films were frozen in liquid nitrogen for several minutes and fractured perpendicularly to their surface. The samples were covered with a carbon layer (5 nm) by a sputtering system (Gatan PECSPIPS, Pleasanton, CA, USA).

#### 2.3.3. Wide- and Small-Angle X-Ray Diffraction Study

The crystalline structure of the films was studied by wide-angle X-ray diffraction (WAXD) with a D8 DISCOVER diffractometer (Bruker, Rheinstetten, Germany) using Cu-K_α_ radiation. Scattering angles varied from 5° to 40°. Small-angle X-ray scattering (SAXS) experiments were performed with “SAXSessmc2” (Anton Paar, Graz, Austria) and Rigaku SmartLab 3 (Rigaku Corporation, Tokyo, Japan) diffractometers.

#### 2.3.4. Thermogravimetric Analysis (TGA)

Thermal properties of the materials were investigated using a DTG-60 setup (Shimadzu, Kyoto, Japan). Samples (approximately 5 mg) were heated up to 600 °C at a rate of 5 °C/min. The experiments were carried out in argon flow (80 mL/min). The TGA curves obtained were used to determine the thermal stability indices, τ_5_ (the temperature value at which a polymer or a composite loses 5% of its initial weight because of the thermal destruction processes), of the chitosan in each sample.

#### 2.3.5. Thermo-Mechanical Analysis

Thermo-mechanical analysis (TMA) was performed using a TMA 402 F1 Hyperion thermal analyzer (NETZSCH, Selb, Germany) with stress applied 0.5 MPa and heating rate 5 °C/min (starting temperature was −75 °C). The experiments were carried out in argon flow (70 mL/min).

#### 2.3.6. Sorption Isotherms

The water sorption isotherms were measured gravimetrically at room temperature using films dried under vacuum at 50 °C. The dried samples were kept at room temperature in desiccators with relative humidities (RH) 17%, 34%, 54%, 76%, and 97% until reaching the constant mass. The mass of absorbed water was calculated and used for the plotting of sorption isotherms. The Laatikainen-Lindström model [38] was used for the fitting of sorption data with Equation (1):

(1) $$a=\frac{a_{m}\alpha h}{\left(1-\beta h\right)\left[1+\left(\alpha -\beta \right)h\right]}$$

where *a_m_* and *h* are the concentration of primary sorption centers and the ratio of the partial pressure to the saturated vapour pressure (*p*/*p*_s_). *a_m_*, α and β are parameters adjusted during the fitting of experimental data.

The Laatikainen–Lindström model describes the sorption of water in a swellable polymer as quasi-chemical multi-stage process. Parameters α and β are connected with the equilibrium constants of the swelling and sorption processes. As a result, the model gives the possibility to calculate amounts of water sorbed on the active primary sorption cites (*a_psc_*) and in clusters (*a_clusters_*) using *a_m_*, α and β parameters. The following equations are used for this purpose:

(2) $$a_{psc}=\frac{a_{m}\alpha h}{1+\left(\alpha -\beta \right)h}$$

(3) $$a_{clusters}=\frac{a_{m}\alpha \beta h^{2}}{\left(1-\beta h\right)\left[1+\left(\alpha -\beta \right)h\right]}$$

#### 2.3.7. Electrical Properties

The electrical measurements were conducted with Alpha-N High Resolution Dielectric Analyzer (Novocontrol Technologies, Montabaur, Germany) on films with *h* = 20 μm thick sandwiched between two platinum electrodes with the diameter 0.56 cm (*S* = 0.25 cm^2^) in the temperature range 298–323 K. The measurement cell was thermostated with LT-408 (LOIP, Saint Petersburg, Russia) thermostat with an accuracy of 0.1 K. Typical impedance spectra for samples are given in Supplementary Figure S1. The impedance data were registered with an equivalent circuit consisting of a resistor and constant phase element connected in series. The bulk resistance of samples (R), the thickness of electrochemical double layer (λ) and the relaxation time of charge carriers (τ) were found from the electrochemical measurements (see ESI for detailed description of calculation of these parameters; their values are listed in Supplementary Table S1). These parameters were used for calculation of bulk ionic conductivity of the films (σ), diffusion coefficient of ions (*D*), charge carrier mobility (μ) and volume density of mobile charge carriers (*n*) using the equations:

(4) $$\sigma =\frac{h}{RS}$$

(5) $$D=\frac{\lambda^{2}}{\tau }$$

(6) $$\mu =\frac{De}{k_{b}T}$$

(7) $$n=\frac{\sigma }{e\mu }$$

where *k*_b_ is the Boltzmann constant, *T* is the absolute temperature and *e* is the charge of an ion.

Additionally, the activation energy of ionic conductivity (*E_a_*) for CS films with different NADES content was found from the dependency of logarithm of conductivity on the reciprocal temperature using the Arrhenius equation:

(8) $$\sigma (T)=\sigma_{0}e^{-E_{\alpha }/RT}$$

where *σ*_0_ is the conductivity at room temperature and *R* is the universal gas constant.

## 3. Results and Discussion

### 3.1. Fourier Transform Infrared Spectra

**Figure 2:**
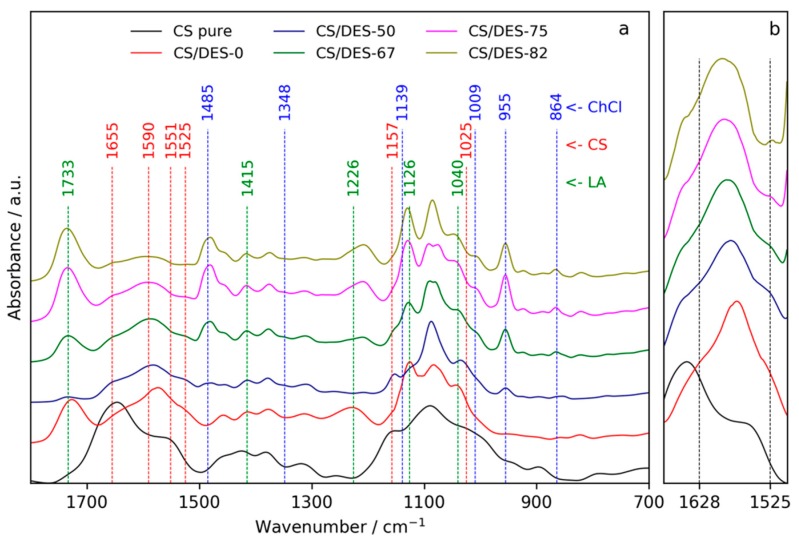
FTIR spectra of the pure CS, CS-LA film (CS/DES-0) and films with different NADES contents (50, 67, 75 and 82 wt%) in the regions cm^−1^ 1800–700 (**a**) and 1680–1500 cm^−1^ (**b**).

The FTIR spectra of pure CS (powder), a film prepared with LA (CS/DES-0) and films prepared with different NADES content are shown in Figure 2a. The difference between spectra of pure CS and CS film containing LA confirms the conversion of CS from base to salt form because of protonation of –NH_2_ groups of the polymer with LA. Particularly, the amide I band (1655 cm^−1^) is shifted to the lower wavenumber (1628 cm^−1^) and a new band, which is attributed to the vibrations of –NH_3_^+^, appears near 1525 cm^−1^ [39]. Both of these bands are seen as shoulders on the red curve in Figure 2b. Characteristic bands of LA [40] at 1733, 1415, 1226, 1126 and 1040 cm^−1^ can also be noticed in the spectrum of CS/DES-0 sample. The addition of ChCl results in the appearance of peaks near 1485, 1009, 955 and 864 cm^−1^, which are at the same positions as for the pure ChCl in the NIST database [41]. At the same time, some characteristic peaks of ChCl (at 1348 and 1139 cm^−1^) did not appear in the spectra of plasticized CS films. The disappearance of these ChCl peaks was observed in the FTIR study of DES malonic acid/ChCl [35]. Thus, it can be considered as an evidence for the formation of NADES inside the CS film.

It can be noticed, that in the case of CS/DES-50 film, the peak corresponding to the –COOH groups (1733 cm^−1^) is not observed, which is not the case for samples with higher NADES content. Disappearance of this band from a carboxylic acid FTIR spectrum takes place in the case of deprotonation of the acid resulting in formation of carboxylate (–COO^−^) group. Taking into account that the content of LA is minimal in CS/DES-50 sample in comparison with the other films, it can be supposed, that in CS/DES-50 film the most part of LA is connected with CS via salt bonding. This hampers the interaction between ChCl and LA. ChCl-LA interactions are enhanced with an increase in the NADES content in the film up to 67 wt% and more. The shifting of band near 1226 cm^−1^, which is a characteristic feature of LA in the CS/DES-0 film, toward lower wavenumbers (1207 cm^−1^) in the films with the addition of ChCl can be also considered as an evidence for the formation of NADES inside films with content of DES > 50 wt%. The same shifting of this acid related peak was observed in the case of the formation of DES malonic acid/ChCl [35].

### 3.2. Scanning Electron Microscopy

**Figure 3:**
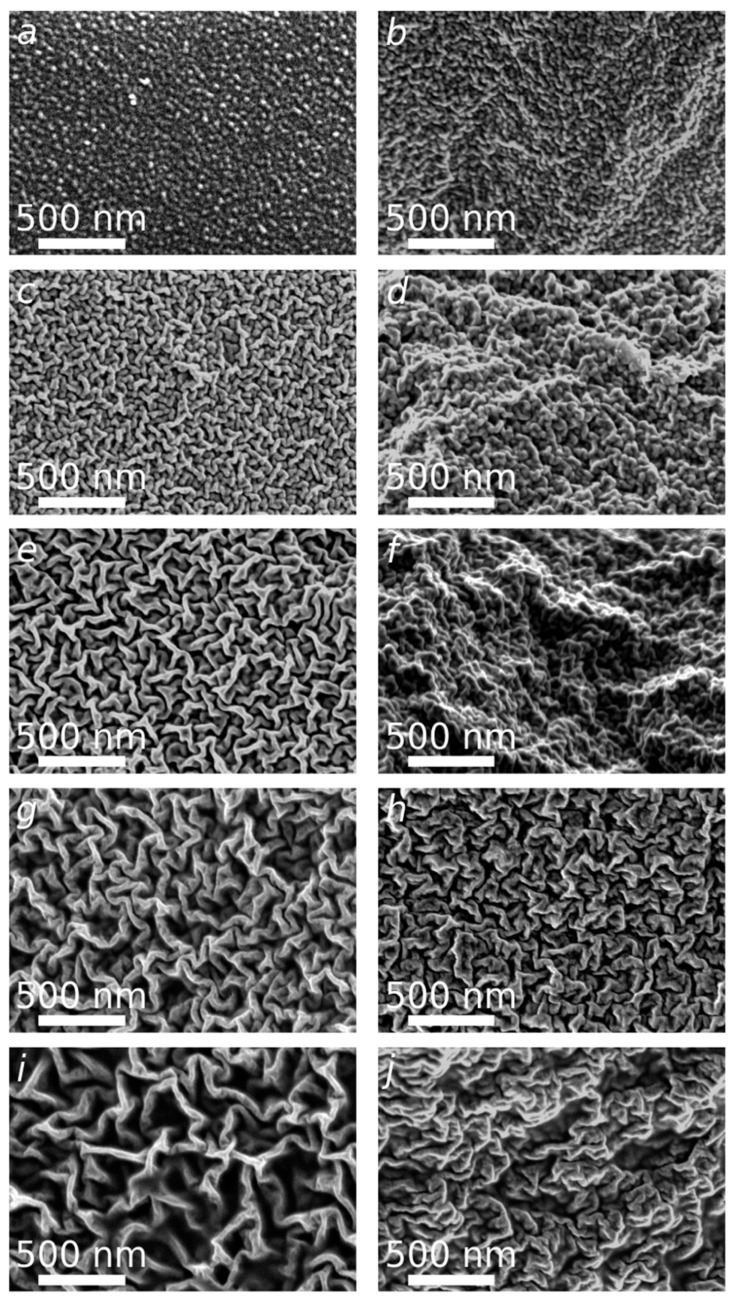
SEM micrographs of the reference CS film (CS/DES-0) (**a**,**b**) and films with different NADES contents: 50 wt% (**c**,**d**), 67 wt% (**e**,**f**), 75 wt% (**g**,**h**) and 82 wt% (**i**,**j**). The surface (**a**,**c**,**e**,**g**,**i**) and the cross-sectional morphology (**b**,**d**,**f**,**h**,**j**) of films.

In order to get more insights into the films structure depending on the NADES content, SEM micrographs of the surface and the cross sections were acquired (Figure 3). The surface of the reference CS film (CS/DES-0) shows high uniformity, smoothness, and grain-like morphology (Figure 3a,b). The incorporation of DES leads to the formation of rough surface. The random orientation of a hierarchical wrinkle structure is observed (Figure 3c,e,g,i). The increase of NADES content from 50 wt% to 82 wt% leads to the gradual increase of wrinkles’ size from about 40 nm to 100 nm in diameter and appearance of free space between the wrinkles. At low NADES content (50, 67%), the wrinkled structure of cross-sections is less pronounced (Figure 3d,f) in comparison with the surface (Figure 3c,e). For the samples with higher NADES content this is not the case: the morphology of cross-sections and surfaces is similar (Figure 3g–j). However, the folds have a slightly more compact structure in terms of volume (Figure 3h,j).

### 3.3. X-Ray Diffraction Investigation

#### 3.3.1. WAXD Data

**Figure 4:**
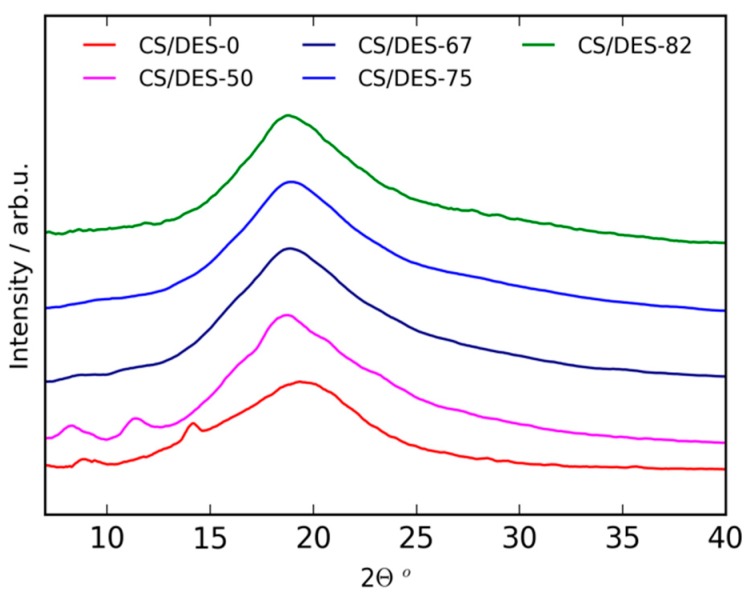
WAXD patterns of CS-LA film (CS/DES-0) and films with different NADES contents (50, 67, 75 and 82 wt%).

The crystalline structure of the samples was investigated with WAXD. The patterns are demonstrated in Figure 4. Values of interplanar distances (*d*) were calculated from the Bragg angles (2θ) of the reflections according to Bragg equation 2*d*Sinθ = nλ, where λ is an X-ray radiation wavelength, *n* is an integer. The WAXD pattern of CS/DES-0 film prepared without ChCl demonstrated reflections at 2θ = 9.2° (9.65 Å), 14.4° (6.19 Å) and 19.6° (4.59 Å). These peaks are typical for CS crystal structure in an anhydrous polymorph [42,43,44,45,46]. It has been considered that in the case of monocarboxylic acids the spontaneous water-removing from their chitosan salt occurs [47]. This is also in agreement with water sorption measurements, presented in Section 3.5., which demonstrate that the CS/DES-0 film has a lower water sorption ability than any sample containing NADES. The incorporation of 50 wt% NADES resulted in the change of peaks positions to 2θ = 8.42° (10.52 Å), 11.33° (7.84 Å) and 18.85° (4.77 Å) revealing that the introduction of 50 wt% NADES caused an emergence of hydrated polymorph of CS [44,46,48,49]. A further increase in the NADES content up to 67 wt% leads to the separation of CS chains due to the formation of NADES inside a film and to amorphization of the samples. The above results well correspond with FTIR data (see Section 3.1.), which demonstrates that 50 wt% NADES is not enough to form NADES inside the film due to the interaction of CS with LA to form the CS salt.

#### 3.3.2. SAXD Data

**Figure 5:**
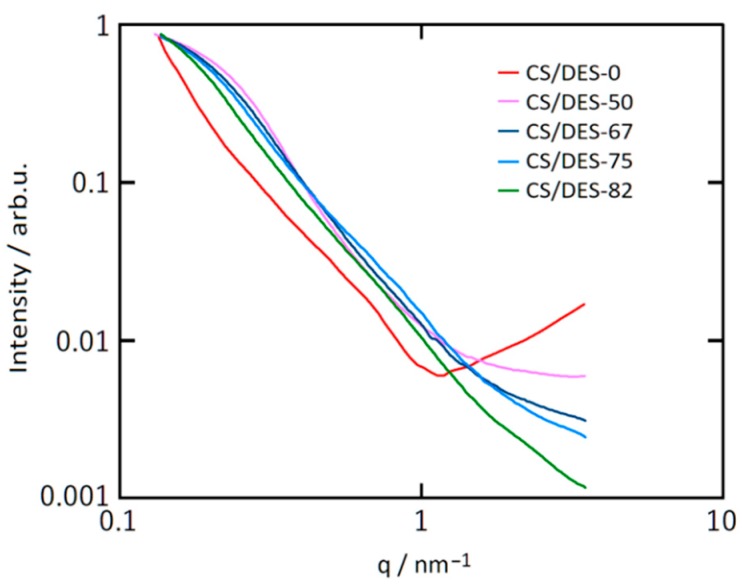
SAXS patterns of CS-LA film (CS/DES-0) and films with different NADES contents (50, 67, 75 and 82 wt%).

The intensity of small-angle X-ray scattering was measured in the range of the scattering vector *q* = 0.13 nm^−1^ … 3.5 nm^−1^ ($q=\frac{4\pi }{\lambda }\sin(\theta )$, where λ is the radiation wavelength and θ is the half of the scattering angle). The obtained curves are presented in Figure 5 on a double logarithmic scale.

**Figure 6:**
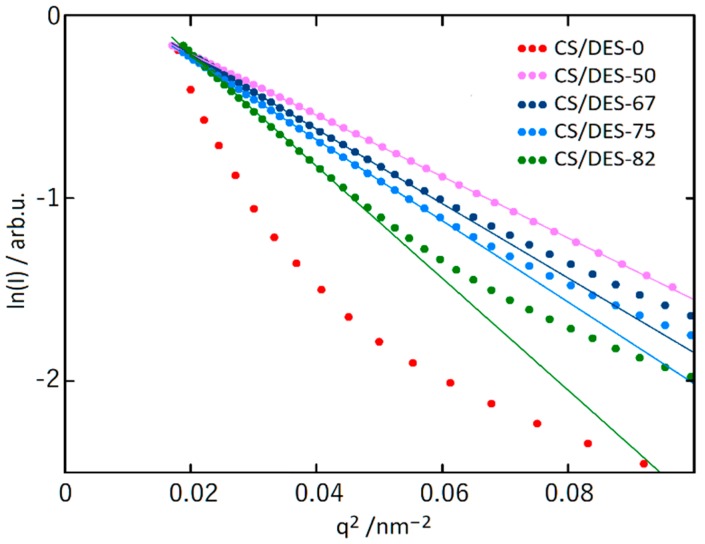
The SAXS patterns for CS-lactic acid film (CS/DES-0) and films with different NADES contents (50, 67, 75 and 82 wt%) in the Guinier coordinates. Straight lines demonstrate the slope of curves at the low *q*^2^ values.

The addition of ChCl, leads to appearing of a hump at low scattering angles for the sample CS/DES-50, that is not observed for CS/DES-0. This hump shifts toward lower *q* values with increasing DES content. Such a behaviour of the intensity indicates the appearance of inhomogeneities of electron density, the average size of which increases with increasing of NADES content. Moreover, the scattering intensity at high *q* values decreases with increasing of NADES content, which can be attributed to a decrease in the relative fraction of small inhomogeneities. The size of inhomogeneities (R_g_) can be estimated from the slope of the initial portion of scattering curves plotted in Guinier coordinates (ln(*I*) vs *q*^2^) [50], which are presented in Figure 6. The following equation is used for this purpose:

(9) $$R_{g}\;=\;\sqrt{-3\frac{\Delta \left(\ln\left(I\right)\right)}{\Delta \left(q^{2}\right)}}$$

The linear behaviour in low *q*^2^ region is clearly observed for all the samples containing NADES and this is not the case for the CS/DES-0 film (Figure 6). The size of inhomogeneities (R_g_) are given in Table 1.

It is interesting to mention that for the sample with the highest NADES content (82 wt%), the intensity plot in the double logarithmic scale has an extended linear section (see Supplementary Figure S3) with the slope α = Δln(*I*)/Δln(*q*) = 2.25. In this case, the intensity decreases according to a power law with increasing *q*: *I* ~ *q*^−α^ in the range of *q* = 0.3–1.6 nm^−1^, which correspond to the range of sizes *d* = 3.9–21 nm. The presence of such a dependence may indicate that in the corresponding size range there is a fractal distribution of electron density. Because α ≤ 3 [51], this behaviour is a characteristic of a system with a fractal distribution of mass, and the fractal dimension *D* is equal to α, i.e., *D* = 2.25.

The increase of sizes of inhomogeneities, observed in SAXS experiments is in agreement with microscopic investigations, discussed earlier (see Section 3.2.). It can be supposed that this increase is connected with “swelling” of chitosan macromolecules in NADES, which is also evidenced by the decreasing of crystallinity, inferred from WAXS results (see Section 3.3.1.). This supports the idea about intensive interaction of CS with DES based on ChCl and LA.

The swelling of CS chains in NADES presumed from X-ray diffraction study can lead to the increasing of gaps between polymer chains. As a result, the diffusion coefficient of ionically conducting species increases and the activation energy of ionic conductivity decreases, which will be demonstrated further.

### 3.4. Thermal Properties of Films

#### 3.4.1. Thermal Gravimetric Analysis

**Figure 7:**
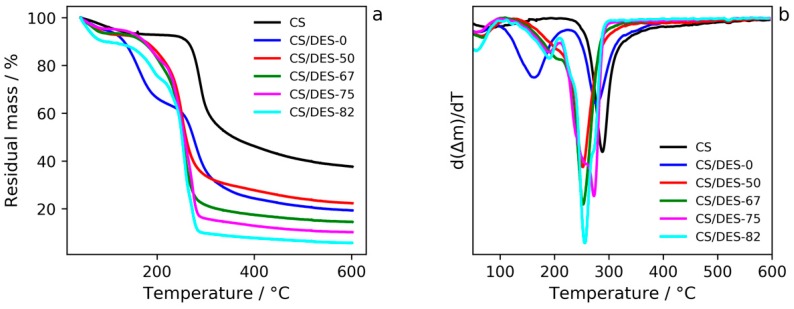
TGA (**a**) and DTG (**b**) curves for CS, CS-LA film (CS/DES-0) and films with different NADES contents (50, 67, 75 and 82 wt%).

It is clearly seen from the TGA curves (Figure 7 and Supplementary Figure S4) that the weight loss, which is likely related to the water evaporation, takes place in the temperature range 40–100 °C. The most pronounced weight loss (ca. 10 wt%) corresponds to the sample with the highest NADES content (82 wt%). This is in agreement with water sorption measurements (see Section 3.5.).

Considering the TGA curve of CS/DES-0 one can notice a decrease in τ_5_ value of CS by 14 °C (see Table 2). This indicates interactions between LA and CS. Apparently the former somehow embeds in the system of CS hydrogen bonding and forms polysalts through attachment to NH_2_-groups of the polymer. The middle step on the TGA curve (Figure 7) may correspond to the destruction process of the LA excess. The latter can be attributed to the amount of LA which was not involved in the aforesaid interactions.

Incorporation of a hydrogen bond acceptor ChCl in the mixture of hydrogen bond donors CS/LA leads to the formation of a quasi-three-component compound whose thermal degradation starts at temperatures much lower (by ~40 °C) than that of pristine CS. This process is reflected in one-step weight loss in the CS/DES-50 and CS/DES-67 curves and points at a quite strong interaction of the polymer with DES. The emergence of an additional step in the middle temperature range for systems CS/DES-75 and CS/DES-82 could probably be assigned to a destruction of NADES excess, the latter being likely a consequence of over-saturation of CS with NADES. Interesting to note, that neither τ_5_ value for CS, nor starting temperature of the NADES degradation depend on the NADES content for CS/DES-75 and CS/DES-82 systems. Moreover, τ_5_ value for CS is almost the same for all of the samples containing NADES (~230 °C) (Table 2).

#### 3.4.2. Thermo-Mechanical Analysis

**Figure 8:**
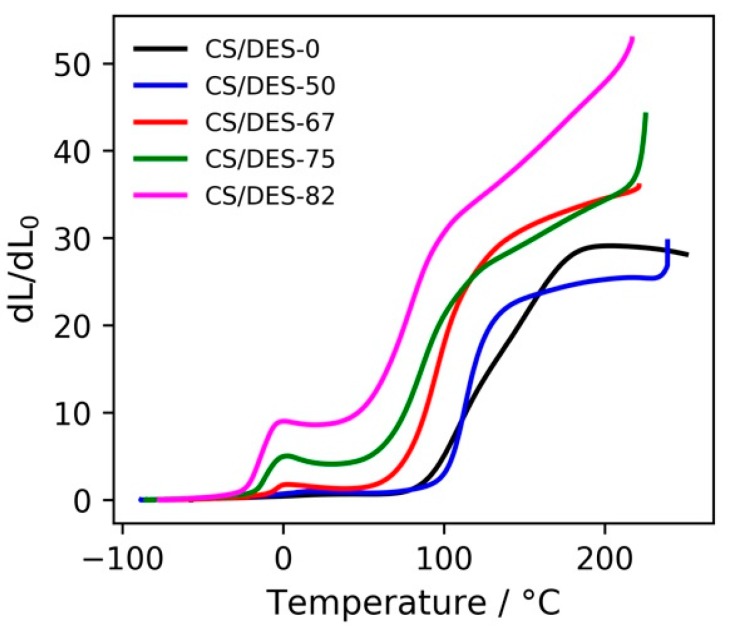
TMA curves for CS—LA film (CS/DES-0) and films with different NADES contents (50, 67, 75 and 82 wt%).

TMA curves (Figure 8) of all of the CS/DES samples are quite similar to those for amorphous polymers and have several regions. The first one (from starting temperature up to about T_g_ values, T_g_ varies from −23 to −5 °C as the NADES content decreases) corresponds to the glass state of the CS, small deformations at moderate stresses applied being observed. This assertion is based on the DSC data obtained in [35] for CS/DES system, which revealed a substantial decrease in T_g_ of the CS (T_g_ ~165 °C) upon mixing with the NADES. Noteworthy is that along with the decrease in T_g_ of the polymer an augment of the film deformation was observed as the NADES content was increased. Obviously, NADES have a plasticizing effect, their molecules being distributed between CS molecules and hindering the inter- and intramolecular interactions between the polymer chains due to formation of strong hydrogen bonds with CS [30]. As a result, the mobility of the macromolecular chains increases. This leads to T_g_ reduction, T_g_ depending on the concentration of the introduced plasticizer.

Considering TGA curves of the samples (Figure 7a) one can notice that even after over-saturation of CS with NADES T_g_ continues decreasing. It could be related to the impact of the NADES excess on the interfaces of relatively large elements of the CS supramolecular structure.

The second region (after glass transition) in the TMA curves (Figure 8) corresponds to the elastic state of the CS, where rather considerable reversible deformations of the polymer occur. The minima on the TMA curves could be related to the entropically driven shrinkage of the polymer chains. Pondering in terms of a balance between the energy of interaction between polymeric chains and the chain entropy, one can presume that the entropic forces evolve, which operate with a result in a sample shrinkage. External stress tends to align neighbouring segments, thereby predetermining rotational constraints along the contour of a chain as a whole with a drop in rotational entropy. On the other hand, one could expect that the external strain brings segments of the chains closer, which would mean entropic gain arising from the free volume effect. The overall effect of the relative shrinkage gives ground to presume a predominance of the rotation entropy loss against the gain in the entropy of the reduced excluded volumes [52,53,54].

The next region (whose onset temperature varies from 54 °C to 102 °C as the NADES content decreases) corresponding to the steep increase in deformation can presumably be asserted to a fluid state of chitosan, movement of polymer chains as a whole probably taking place. It is worth mentioning that such an augmentation of deformation can also be related with fast-evolving reversible elastic deformation caused by high temperature.

### 3.5. Water Sorption Measurements

The application of CS films in the ambient conditions leads to the interaction of material with the moisture and sorption of water in the film. Water can significantly influence the mechanical and transport properties of the prepared materials by changing the internal structure. Thus, analyzing the water sorption ability for the investigated films is needed and the state of water inside a film is significant for appropriate discussion of other properties. It needs to be mentioned that water can act as an additional plasticizer for CS. It is significant for this work to understand at what values of water vapour activity the plasticizing effect of water should be taken into account.

**Figure 9:**
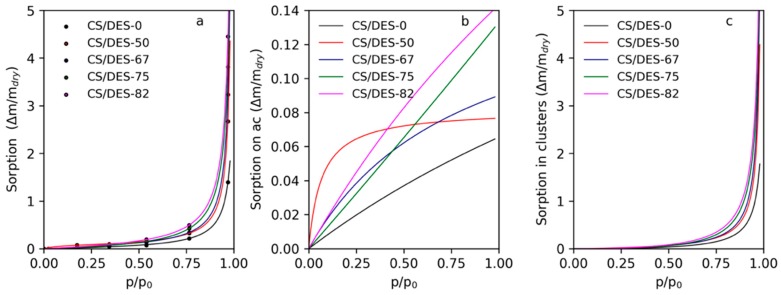
Water sorption isotherms for CS film prepared with LA and with different concentrations of LA-ChCl NADES (**a**); sorption on active sorption centers (**b**) and in clusters (**c**).

The experimental results of water sorption at different RH for CS film prepared with LA without ChCl and with addition of different amounts of NADES are shown in Figure 9a with circle markers. The curves in this plot demonstrate the Laatikainen–Lindström isotherms with parameters fitted according to Equation (1): the values of found parameters are listed in Table 3. It is seen that the amount of active sorption sites increases gradually with increasing of content of ChCl. This means that ChCl gives significant impact to the sorption of water on primary sorption centers in the CS/DES films. At the same time the activity of primary sorption centers toward binding of water is maximal for CS/DES-50 film and decreases either on the elimination of ChCl or on the decreasing of CS content. It can be proposed that in comparison with the CS/DES-0 sample, the CS/DES-50 film contains a lower amount of the LA, which is partly incorporated in the interaction with ChCl. As a result, a high amount of free -OH and -NH_2_ groups can be expected. These groups can act as primary sorption centers with high activity as was demonstrated earlier [35]. The increase of the NADES content leads to the decreasing of α value, which is illustrated as decreasing of curvature of the curves of sorption on active centers, given in Figure 9b. This reflects changes in the nature of primary sorption centers because of decreasing of CS content and increasing of ChCl content.

The parameter β reflects the absorption of water in the material due to binding with secondary centers, i.e., water molecules connected to the active sorption sites in a sorbent. Also, this parameter depends on the mobility of polymer chains that is needed for the formation of “free space” near the water adsorbed on a primary sorption center. Overall, this parameter demonstrates the formation of water molecule clusters inside the sorbent. This parameter varies from 0 to 1 and the higher values mean the higher tendency toward cluster formation. In the case of CS films with lactic acid the value of β is very close to 1. The increasing of β with addition of ChCl is connected with the increasing of the mobility of CS chains because of the NADES formation inside the film, which leads to the plasticizing of the polymer as it was shown with mechanical measurements. The predicted part of water absorbed in clusters can be found from the parameters of the Laatikainen-Lindström isotherm and is given in Figure 9c. It is seen that up to values of RH 50%–60% the amount of water sorbed in clusters is insignificant. It can be proposed that at RH < 50% water does not increase the mobility of polymer chains enough for the formation of additional “free space” needed for clusters formation. Thus, the additional plasticizing effect of water is expected at RH > 50%.

### 3.6. Mechanical Properties

**Figure 10:**
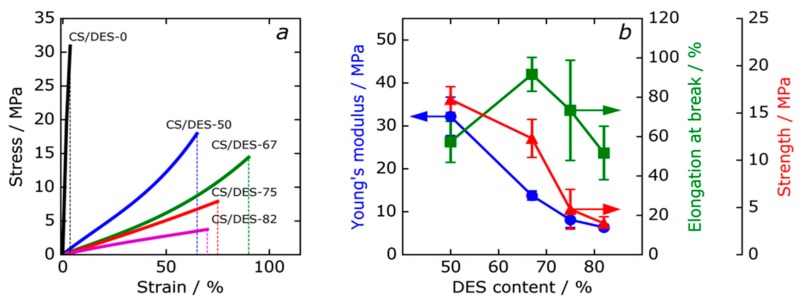
Typical view of stress-strain curves for the reference CS film (CS/DES-0) and composites with different NADES contents (**a**). Dependences of mechanical properties of the films on the NADES content (**b**).

Figure 10a shows typical stress-strain curves of the CS film (CS/DES-0) and the CS/DES films. The effect of the NADES content on the mechanical properties of the composite films is shown in Figure 10b. Young’s modulus, elongation at break and strength of the composite films are summarized in Table 4. An increase of the NADES content led to a decrease of Young’s modulus from 32 MPa for CS/DES-50 film down to 6 MPa for CS/DES-82 (Figure 10b). At the same time, the elongation at break increases from 60% to 92% when the amount of NADES increase from 50 to 67 wt% (Figure 10b), which is connected with an increase in the molecular mobility of the polymer. It should be noted that the CS prepared without ChCl demonstrated 3.5% of elongation at break. This proves that it is the combination of LA and ChCl that leads to the plasticizing effect on the CS film. It needs to be noticed, that the water sorption ability of CS film containing NADES is higher than that of CS/DES-0 (see Section 3.5). Thus, in the case of LA-ChCl NADES the plasticizing effect can also be facilitated by water. Further increasing of the DES content up to 75 and 82 wt% leads to a reduction of elongation at break, which can be explained by the destruction of intermolecular hydrogen bonds between the CS chains due to the interaction of the −OH groups of the polymer with the NADES components and water. The results of the mechanical measurements agreed with the observations of cross-section with SEM, which showed a change of character of the fracture surface at the increase of the NADES content from 67 to 75 wt%.

### 3.7. Ionic Conductivity and Transport Properties of CS/DES Films

Ionic conductivities of prepared films measured at room temperature are listed in Table 5. The CS/DES-0 film prepared without ChCl demonstrates the ionic conductivity 2.8 × 10^−5^ mS/cm. The addition of NADES leads to the gradual increase in ionic conductivity up to 1.7 mS/cm for the films containing 82 wt% of NADES. This value is comparable to the conductivity of the pure LA-ChCl NADES with low water content [37], which along with results of other methods confirms the successful formation of NADES inside CS films.

**Figure 11:**
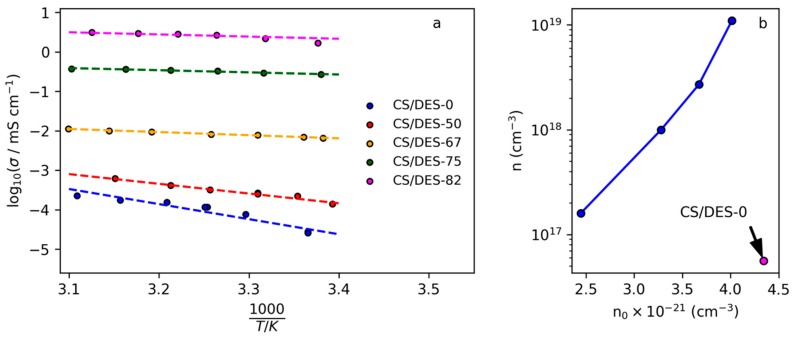
(**a**) Dependences of logarithm of electrical conductivity on the reciprocal temperature for the film CS with LA and CS films prepared with different NADES content (*a*) and (**b**) dependence of the number density of charge carriers (*n*) on the number density of -COOH groups (*n_0_*) in the films.

The linear dependences of the logarithm of conductivity on the reciprocal temperature (Figure 11a) give possibility to find the activation energy of ionic conductivity for the prepared films. It can be noticed that the most significant change in *E*_a_ and σ takes place when the concentration change from 50 to 67 wt% of NADES, which correlates with significant changing in diffusion coefficient and mobility of ions (see Table 5). This result correlate with data on mechanical properties, which demonstrate the significant increasing of elongation at break when the NADES content increases from 50 to 67 wt%. This can be attributed to the significant increasing of the polymer chain mobility and is in agreement with FTIR results, which show that 50 wt% is insufficient for the formation of NADES inside film because of concurrent interaction of CS with LA with formation of CS salt.

For the samples with NADES content from 67 up to 82 wt% the diffusion coefficient, activation energy and charge carrier’s mobility remain within the same order of magnitude each (increasing 2 about times), while electrical conductivity continues gradual growth. This is connected with increasing of density of charge carriers: for the CS/DES-82 the value of *n* is more than 10 times higher than that for the CS/DES-67 sample (Table 5). Thus, it can be concluded that growing of conductivity at the NADES content < 67 wt% is connected with plasticizing of the polymer, while at NADES content > 67 wt% with increasing density of charge carriers. It is interesting to notice that the value of E_a_ for the films with the high NADES content (10 kJ/mol = 2.4 kcal/mol) is in the range proposed for Grotthuss mechanism of proton transport (2–3 kcal/mol) [55]. Thus, this mechanism can be suggested for the movement of H^+^ in films with NADES content > 67 wt%.

It can be assumed, that mobile charge carriers in the investigated system are H^+^ ions from –COOH groups of LA. In this sense it is interesting to analyze the influence of concentration of LA in films on the concentration of charge carriers. As shown in Figure 11b, the CS/DES-0 film contains maximal amount of LA, however the number of charge carriers is minimal. The addition of ChCl leads to the decreasing of concentration of LA in the films and increasing of concentration of mobile charge carriers due to the plasticizing effect. Increasing of NADES content leads to the exponential growth of density of charge carriers. This can be explained by the partial binding of LA with CS, which was demonstrated by FTIR. At 50 wt% of DES the most part of LA is connected to CS, leaving a small amount of –COOH groups for generation of mobile H^+^ ions. At the same time, the capability of CS toward strong binding of the acid is limited by the number of –NH_2_ groups. Thus, at NADES concentrations higher than 50 wt%, the addition of a greater amount of LA leads to the nonlinear growth of the acid not bonded to the polymer.

## 4. Conclusions

The ionically conducting films based on the chitosan and choline chloride, i.e., lactic acid natural deep eutectic solvent (NADES), were prepared with the NADES content up to 82 wt%. It was demonstrated by a combination of FTIR, scanning electron microscopy, wide-angle and small-angle X-ray scattering, that components of NADES intensively interact with the polymer, that results in the “swelling” of polymer chains and decreasing of the degree of crystallinity. At the same time, the formation of NADES inside the film is proposed to begin at NADES content 67 wt%, while at lower concentrations, the lactic acid interacts mostly with chitosan. The strong NADES-polymer interaction leads to the appearance of two transitions at temperatures between −23 and −5 °C and 54–102 °C depending on the NADES content. The maximal ionic conductivity is achieved for the film with 82% of the NADES content −1.7 mS/cm, which is comparable with reported values of choline chloride—lactic acid NADES. It was demonstrated, that addition of choline chloride significantly increases the mobility and number of charge carriers in the film even at smaller acid concentration in the membrane. The increasing of the NADES content from 50 to 82 wt% leads to the exponential growth of concentration of mobile charge carriers and increasing of their diffusion coefficient. This is connected with the swelling of polymer chains in NADES and with decreasing of crystallinity of the film. The obtained results can be significant for the possible application of NADES–chitosan based films as ion gel separators, electrolytes for organic electronics, or signal transducers.

## Figures and Tables

**Table 1:** The size of inhomogeneities (R_g_) estimated from the slope of the initial portion of scattering curves plotted in Guinier coordinates.

Sample	R_g_, nm
CS/DES-50	7.1
CS/DES-67	7.8
CS/DES-75	8.2
CS/DES-82	9.6

**Table 2:** TGA results of decomposition process of samples.

Sample	τ_5_, °C	τ_5_, °C *
CS	-	269
CS/DES-0	134	255
CS/DES-50	178	228
CS/DES-67	189	228
CS/DES-75	172	229
CS/DES-82	169	232

*: τ_5_ was calculated according to the stage of the most intense step weight loss.

**Table 3:** Refined parameters of the Laatikainen-Lindström isotherm, for CS film prepared with LA without addition of ChCl and with different NADES content.

NADES Content, wt%	*a_m_*, g/g	α	β
0	0.07	1.28	0.98
50	0.08	15.85	1
67	0.09	2.23	1
75	0.13	0.96	1
82	0.14	1.37	1

**Table 4:** Mechanical properties of CS film with LA (CS/DES-0) and films with different NADES content.

Sample	Modulus, MPa	Strength, MPa	Elongation at Break, %
CS/DES-0	1300 ± 100	31 ± 2	3.5 ± 1
CS/DES-50	32 ± 5	16 ± 1	60 ± 10
CS/DES-67	14 ± 2	12 ± 2	92 ± 9
CS/DES-75	8 ± 2	5 ± 2	75 ± 25
CS/DES-82	6 ± 1	3.3 ± 0.7	50 ± 14

**Table 5:** The values of room temperature ionic conductivity (σ), diffusion coefficient of ions (*D*), mobility (*μ*) and number density of charge carriers (*n*) and activation energy of ionic conductivity (*E_a_*) for CS film with LA and films with different NADES content.

Sample	σ, mS/cm	*D*, cm^2^/s	*μ*, cm^2^/V s	*n*, 1/cm^3^	*E_a_*, kJ/mol
CS/DES-0	2.8 × 10^−5^	8.1 × 10^−8^	3.2 × 10^−6^	5.6 × 10^16^	73
CS/DES-50	1.4 × 10^−4^	1.4 × 10^−6^	5.7 × 10^−5^	1.6 × 10^17^	47
CS/DES-67	6.6 × 10^−2^	1.0 × 10^−5^	4.1 × 10^−4^	1.0 × 10^18^	15
CS/DES-75	2.7 × 10^−1^	1.6 × 10^−5^	6.4 × 10^−4^	2.7 × 10^18^	10
CS/DES-82	1.7 × 10^0^	2.3 × 10^−5^	9.2 × 10^−4^	1.1 × 10^19^	10

## Supplementary Materials

The following are available online at https://www.mdpi.com/2073-4360/12/2/350/s1, Table S1: Parameters of equivalent circuit and electrochemical double layer for CS films prepared with LA (CS/DES-0) and with different DES content, Figure S1: Impedance spectra measured at 298 K (a, d, g, j, m), dependences of loss tangent on frequency (b, e, h, k, n) and dependences of real component of dielectric permittivity on frequency (c, f, i, l, o) for CS/DES-0 (a-c), CS/DES-50 (d-f), CS/DES-67 (g-i), CS/DES-75 (j-l), CS/DES-82 (m-o) films. Blue points represent experimental results, while green crosses demonstrate the points calculated with fitted parameters, Figure S2: SEM micrographs of the reference CS film (CS/DES-0) (a, b) and films containing 50 wt% (c, d), 67 wt% (e, f), 75 wt% (g, h) and 82 wt% (i, j) of NADES, observed with different magnification. The surface (a, c, e, g, i) and the cross-sectional morphology (b, d, f, h, j) of films, are presented, Figure S3: SAXS patterns of CS/DES-82. Extended linear section is marked by squares, Figure S4: TGA curves in temperature range 40–200 °C.
