# Supplementary material for: Ionic Conductivity and Structure of Chitosan Films Modified with Lactic Acid-Choline Chloride NADES
Source: Polymers (Basel). 2020 Feb 6;12(2):350. doi: 10.3390/polym12020350 (PMC7077437; doi:10.3390/polym12020350)
Supplement: Supplementary file 1 [file polymers-12-00350-s001.pdf]

polymers

Supplementary Materials: Ionic Conductivity and Structure of Chitosan Films Modified with Lactic Acid-Choline Chloride NADES

Mikhail A. Smirnov [1], Alexandra L. Nikolaeva [1], Vitaly K. Vorobiov [1], Natalia V. Bobrova [1], Ivan V. Abalov [1], Alexander V. Smirnov [2] and Maria P. Sokolova [1,3,*]

Determination of transport properties of CS-DES films

The transport properties of CS-DES membranes were found from the results of impedance measurements, which are given in Figure S1a, d, g, j and m for CS film prepared with LA and CS films containing 50, 67, 75 and 82 wt% of NADES respectively. The measurements were performed with Alpha-N High Resolution Dielectric Analyzer (Novocontrol Technologies, Germany) on films with 20 μm thick sandwiched between two platinum electrodes with diameter 0.56 cm. The impedance curves demonstrate the linear growth in the low frequency region, which is attributed to the capacitance of double layer. Because of the deviation of growth from vertical direction, this capacitance can be described with constant phase element (CPE). The depressed semicircle in the high frequency region corresponds to the geometrical capacitance of the cell and to the volume ionic conductivity of the material. It can be proposed, that at low frequency region the geometrical capacitance does not influence the measured impedance, thus the equivalent circuit can be simplified as the serial connection of CPE for capacitance of the double layer and resistor for ionic conductivity. The impedance of this scheme can be expressed with the following equation:

$$Z = R + \frac{1}{Q(i\omega)^{\alpha}} \quad (1)$$

where R is the bulk resistivity of the material, Q and α are the parameters of CPE, ω is the angular frequency and i is the imaginary unit. The experimental region of low frequencies was fitted using the Python 3.6 and module scypi. The found parameters of equivalent circuit are given in Table S1. The estimated values of real and imaginary part of impedance found using parameters of fitting are shown with green markers in Figure S1a, d, g, j and m. The good correlation with experimental results (shown with blue circles) demonstrates the successful fitting.

The capacitance of double layer (C_{dl}) can be calculated according to approach described by G.J. Brug at al. [1] with the following equation:

$$Z = R + \frac{1}{Q(i\omega)^{\alpha}} \quad (2)$$

Then, its value can be used for calculation of thickness of double layer (λ):

$$Z = R + \frac{1}{Q(i\omega)^{\alpha}} \quad (3)$$

where ε_r and ε_0 are the relative dielectric permittivity of the sample and dielectric permittivity of vacuum respectively and A is the surface area of electrodes.

The value of ε_r was found by fitting of dependences of real part of dielectric permittivity (ε') on the current frequency according to equation given in [2] for system with blocking electrodes:

$$\varepsilon' = \varepsilon_r \left(1 + \frac{\delta}{1 + (\omega\tau_2)^{2(1-\alpha)}\delta}\right) \quad (4)$$

where δ is the ratio between half of sample thickness and λ, τ_2 is the relaxation time and α is a constant (α<1). Taking into account that the movement of heavy charged molecules like chlorine and choline can disturb the dependence of ε' on frequency at low frequencies, only the high frequency region was used for fitting. The experimental data and the result of fitting are demonstrated in Figure S1c, f, i, l and o for CS prepared with LA and CS prepared with 50, 67, 75 and 82 wt% of NADES respectively. The found values of ε_r are listed in Table S1.

The characteristic relaxation time of mobile charge carriers (τ) was found from the maximum on the dependences of loss tangent (Figure S1 b, e, h, k and n) on the applied frequency and are listed in Table S1.

Table 1. Parameters of equivalent circuit and electrochemical double layer for CS films prepared with LA (CS/DES-0) and with different DES content.

Sample	Q, Ohm^{-1} s$^{\alpha}$	R, Ohm	α	C_{dl}, F	λ, cm	ε_r	τ, c
CS/DES-0	1.5×10^{-7}	280,000	0.57	1.4×10^{-8}	6.8×10^{-6}	4	5.8×10^{-4}
CS/DES-50	4.4×10^{-7}	5700	0.67	2.3×10^{-8}	2.9×10^{-6}	3	5.9×10^{-6}
CS/DES-67	1.9×1^{-6}	120	0.76	1.6×10^{-7}	2.3×10^{-6}	16	4.7×10^{-7}
CS/DES-75	2.9×10^{-6}	29	0.78	2.2×10^{-7}	1.6×10^{-6}	16	1.6×10^{-7}
CS/DES-82	3.3×10^{-6}	4.8	0.85	4.9×10^{-7}	1.3×10^{-6}	29	7.4×10^{-8}

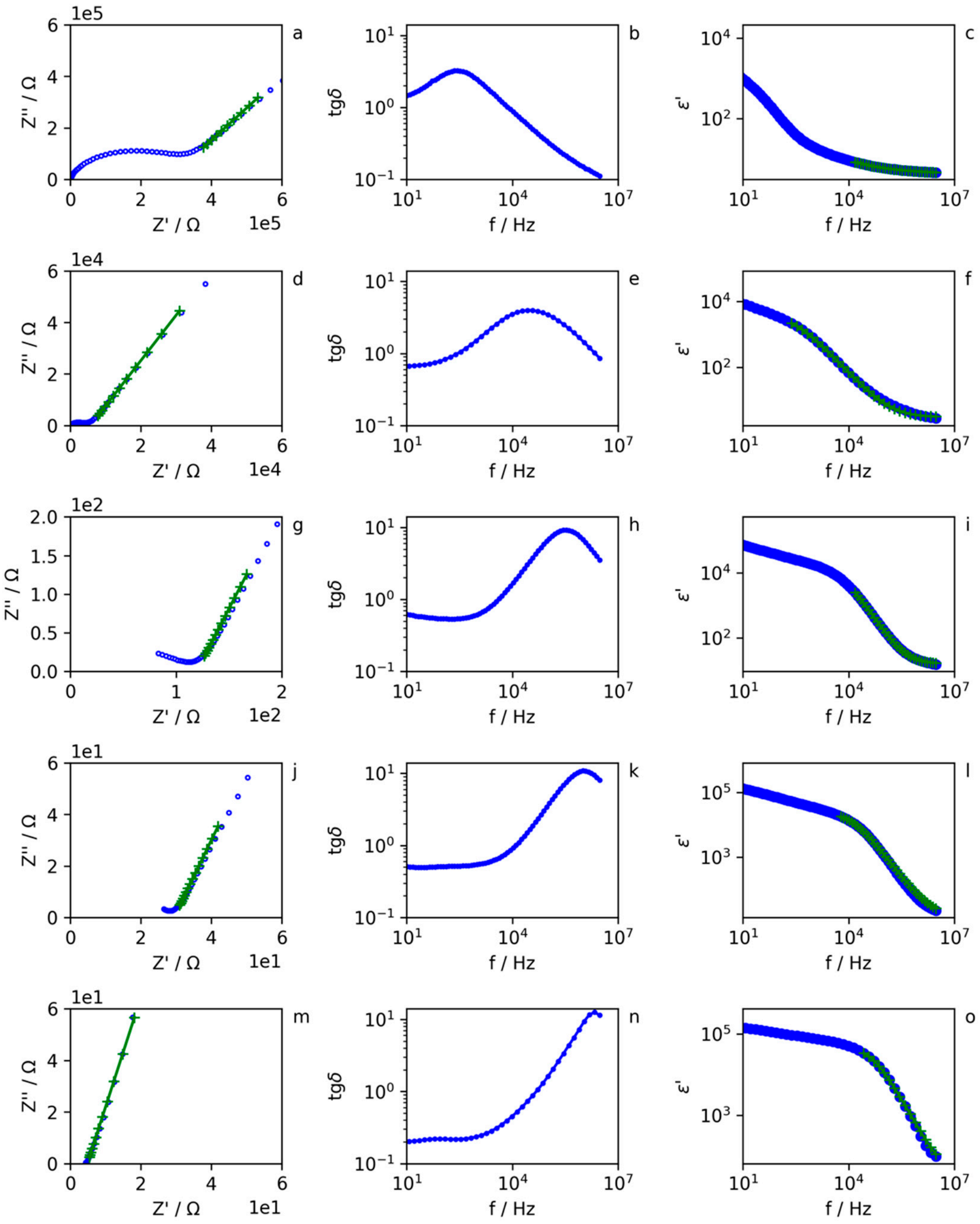

Figure 1. Impedance spectra measured at 298 K (a, d, g, j, m), dependences of loss tangent on frequency (b, e, h, k, n) and dependences of real component of dielectric permittivity on frequency (c, f, i, l, o) for CS/DES-0 (a-c), CS/DES-50 (d-f), CS/DES-67 (g-i), CS/DES-75 (j-l), CS/DES-82 (m-o) films. Blue points represent experimental results, while green crosses demonstrate the points calculated with fitted parameters.

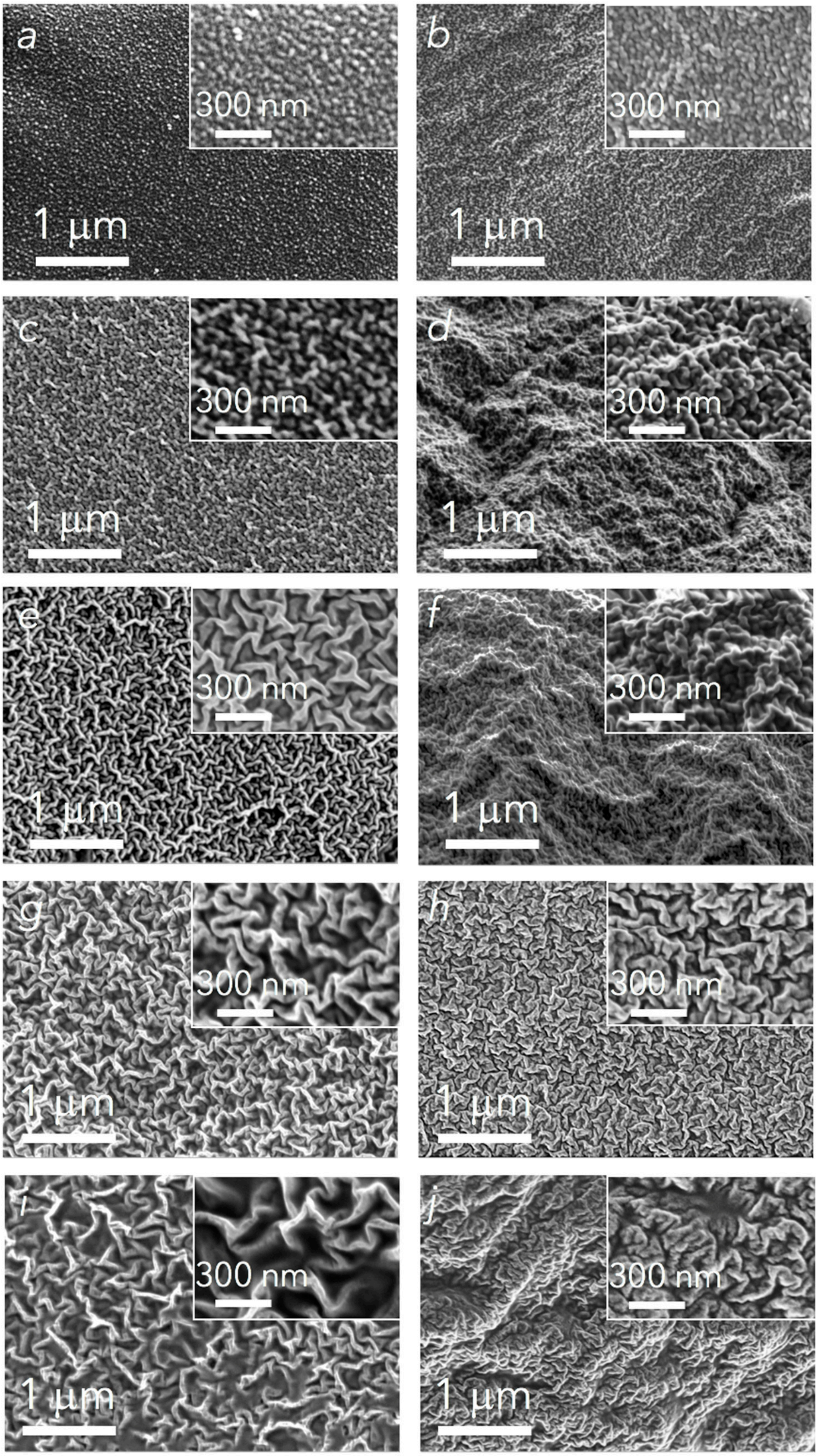

Figure 2. SEM micrographs of the reference CS film (CS/DES-0) (**a**,**b**) and films containing 50 wt% (**c**,**d**), 67 wt% (**e**,**f**), 75 wt% (**g**,**h**) and 82 wt% (**i**,**j**) of NADES, observed with different magnification. The surface (a, c, e, g, i) and the cross-sectional morphology (b, d, f, h, j) of films, are presented.

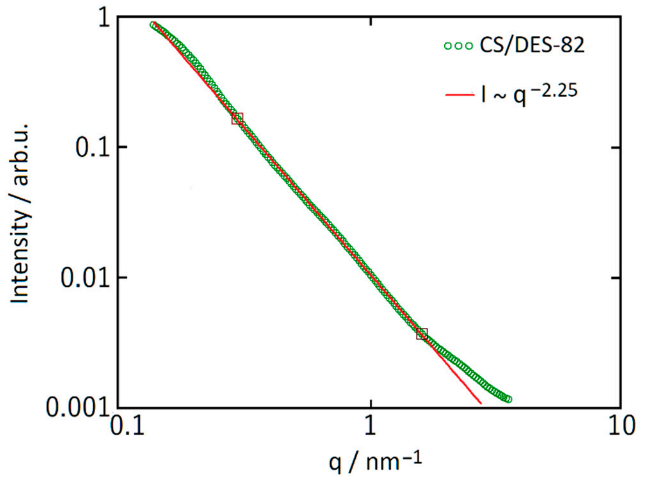

Figure 3. SAXS patterns of CS/DES-82. Extended linear section is marked by squares.

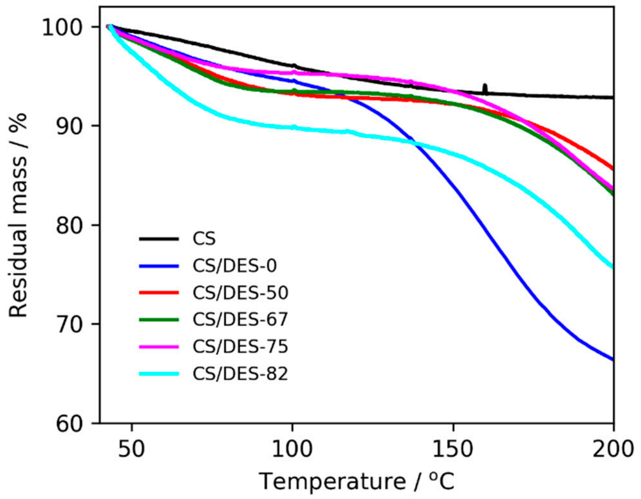

Figure 4. TGA curves in temperature range 40-200 °C.

References

1. Brug, G. J.; van den Eeden, A. L. G.; Sluyters-Rehbach, M.; Sluyters, J. H. The Analysis of Electrode Impedances Complicated by the Presence of a Constant Phase Element. *J. Electroanal. Chem.* **1984** DOI: 10.1016/S0022-0728(84)80324-1.
2. Bandara, T.M.W.J.; Mellander, B.-E. Evaluation of Mobility, Diffusion Coefficient and Density of Charge Carriers in Ionic Liquids and Novel Electrolytes Based on a New Model for Dielectric Response in Ionic Liquids: Theory, Properties, New Approaches; Kokorin, A., Ed.; InTech, 2011, 383–406.

© 2020 by the authors. Licensee MDPI, Basel, Switzerland. This article is an open access article distributed under the terms and conditions of the Creative Commons Attribution (CC BY) license (http://creativecommons.org/licenses/by/4.0/).
